# The Enduring Challenge of Determining Pneumonia Etiology in Children: Considerations for Future Research Priorities

**DOI:** 10.1093/cid/cix143

**Published:** 2017-05-27

**Authors:** Daniel R. Feikin, Laura L. Hammitt, David R. Murdoch, Katherine L. O’Brien, J. Anthony G. Scott

**Affiliations:** 1 International Vaccine Access Center, Department of International Health, Johns Hopkins Bloomberg School of Public Health, Baltimore, Maryland;; 2 Division of Viral Diseases, National Center for Immunizations and Respiratory Diseases, Centers for Disease Control and Prevention, Atlanta, Georgia;; 3 Kenya Medical Research Institute–Wellcome Trust Research Programme, Kilifi;; 4 Department of Pathology, University of Otago, and; 5 Microbiology Unit, Canterbury Health Laboratories, Christchurch, New Zealand;; 6 Department of Infectious Disease Epidemiology, London School of Hygiene & Tropical Medicine, United Kingdom

**Keywords:** pneumonia, etiology, causation, acute lower respiratory tract infections.

## Abstract

Pneumonia kills more children each year worldwide than any other disease. Nonetheless, accurately determining the causes of childhood pneumonia has remained elusive. Over the past century, the focus of pneumonia etiology research has shifted from studies of lung aspirates and postmortem specimens intent on identifying pneumococcal disease to studies of multiple specimen types distant from the lung that are tested for multiple pathogens. Some major challenges facing modern pneumonia etiology studies include the use of nonspecific and variable case definitions, poor access to pathologic lung tissue and to specimens from fatal cases, poor diagnostic accuracy of assays (especially when testing nonpulmonary specimens), and the interpretation of results when multiple pathogens are detected in a given individual. The future of childhood pneumonia etiology research will likely require integrating data from complementary approaches, including applications of advanced molecular diagnostics and vaccine probe studies, as well as a renewed emphasis on lung aspirates from radiologically confirmed pneumonia and postmortem examinations.

Over the past century, findings of pneumonia etiology studies in children have swung from detection of only bacteria to a preponderance of viruses. This apparent change in the microbial etiology of pneumonia is attributable, perhaps, as much to changes in study design and methodology as to true changes in etiology. The same can be said when comparing the results across recent pneumonia etiology studies. Interpretation and comparison of results from studies that use different case definitions, study designs, specimen collection approaches, and diagnostic tests require recognition of the biases inherent in each approach. Yet, the challenges of determining pneumonia etiology extend beyond controlling for bias. The syndrome of pneumonia is inherently challenging to define and diagnose, and its pathogenesis is complex.

In this article, we explore the challenges of determining the microbial etiology of pneumonia, starting with a brief history of pneumonia etiology studies, with particular emphasis on the challenges faced by each era of research. We then enumerate the principal enduring challenges, demonstrating how each challenge can influence results. Finally, we comment on approaches for future research that could resolve the challenges.

## HISTORY OF PNEUMONIA ETIOLOGY STUDIES

### Early Focus on the Pneumococcus

*Streptococcus pneumoniae*, the most important cause of lobar pneumonia, was first identified in 1881 from samples of human saliva. Although it caused “sputum septicaemia” when inoculated into rabbits, *S. pneumoniae* was not recognized as a human pathogen for several years [1, 2]. The first claims for a “micrococcus of pneumonia” were made by Carl Freidländer, who observed diplococci in lung sections from 8 fatal cases in 1882 [3]. In 1883, he cultured “cocci” from animal lung tissue, which were in fact *Klebsiella pneumoniae—*an organism that grew more avidly on the media used and, being short bacilli found in pairs, were confused with the pneumococcus when scientists used methods of the time [4]. In the same year, lung aspirates were first performed in living pneumonia patients, and oval diplococci were observed in the lung exudate [5, 6]. Isolation of these lung diplococci and demonstration of their animal pathogenicity by Albert Fränkel in 1886 led to the proper assignation of etiology and also to the name—pneumococcus [7].

Early pneumonia etiology studies focused on adults, and at that time, pneumococcus was the dominant pathogen. Pneumococcal serotypes were numbered in the order in which they were discovered, and these early serotypes tended to cause epidemics of pneumonia in adults (eg, serotypes 1, 2, 5). Because these “adult epidemic types” rarely colonize the nasopharynx of healthy individuals (unlike most other serotypes), the specificity of sputum culture for pneumonia etiology was high in the early studies among adults. This combination of high pneumococcal prevalence and high etiologic specificity gave sputum culture a useful positive predictive value for pneumococcal pneumonia. In a 1926 series of 2000 adult pneumonia cases from Bellevue Hospital in New York City, pneumococci were found in 95% (mostly sputa) [8]. In a study of 1561 cases at Boston City Hospital in 1933, 98% were attributed to the pneumococcus; 307 of these patients underwent autopsy, and *S. pneumoniae* was cultured from 220 cadavers in either blood or lung material [9]. The prevalence of pneumococcus among cultures from sputum, lung aspirate, and blood from infants and children with pneumonia was slightly lower than in adults but still high; in New York City from 1928 to 1936, pneumococci were found in 923 (53.9%) of 1712 episodes [10].

The development and evaluation of horse serum therapy for pneumococcal pneumonia during the 1920s lent urgency to determining etiology and, if pneumococcal, to identifying the serotype. In general, isolates were obtained from sputum cultures, but it was recognized that lung material, aspirated by percutaneous needles, provided more accurate information and, importantly, may be the only specimen available in young children who swallow rather than expectorate their sputum. In New York, between 1928 and 1936, Bullowa pioneered both lung aspiration and serum therapy, reporting 405 such procedures in children and nearly 1500 in adults [10].

### The Advent of Antibiotics

When sulphonamides and penicillin replaced serum therapy as standard treatment for pneumonia in the 1940s, the incentive to define the infecting organism in individual cases receded, as did the effort to develop pneumococcal vaccines. There are few published studies of pneumonia etiology in the subsequent 30 years. In 1967 the first case of pneumonia caused by a pneumococcus resistant to penicillin was reported in Australia [11], but the clinical and epidemiological significance of this report was not appreciated for many years. In fact, the stimulus to re-examine etiology across the world was the desire to make appropriate life-saving antibiotics more widely available to children to reduce the mortality rate in low-income settings. In the 1980s, the first etiology studies in developing countries were conducted using blood and lung aspirate cultures that focused on bacterial etiologies. In The Gambia, bacteria were cultured in 33 (65%) of 51 children investigated [12]. In Papua New Guinea, 51 (61%) of 83 children had positive cultures; 32 had *Haemophilus influenzae,* and 28 had *S. pneumoniae*, including 10 who had both [13]. The salient feature of these studies was their focus on radiologically evident pneumonia in children with no prior exposure to antibiotics. Indeed, some studies at the time showed that antibiotic treatment of pneumonia in developing countries, using a nonspecific clinical case definition, could reduce mortality, affirming the important role of bacteria in causing severe pneumonia [14].

The World Health Organization (WHO) used these data to develop a policy for case management of acute respiratory illness. They created a clinical case definition for pneumonia not based on radiographic findings, in contrast to the pneumonia etiology studies on which the policy was derived [12, 13]. The WHO clinical case definition for pneumonia deliberately increased sensitivity to ensure that no child with pneumonia should miss the opportunity for effective antibiotic therapy, and this decreased the specificity for lung infection [15]. In the WHO case definitions, “severe pneumonia” was defined by a single clinical characteristic, lower-chest-wall indrawing; “non-severe pneumonia” was defined by tachypnea. Inevitably, by using these definitions for case management, the proportion of children with pneumonia would have been lower and the proportion with a bacterial cause of pneumonia would have been substantially lower than in the studies used to originate the policy.

Early access to antibiotics presented a difficulty for research on pneumonia etiology. Evidence of prior treatment with antibiotics is associated with a 30% reduction in blood culture positivity in children with pneumonia [16], and this may have downgraded the prevailing perception of bacteria as the primary cause of pneumonia. In addition, etiology research in the 1980s and 1990s adopted the WHO clinical management case definitions, which led to the inclusion of many children who did not actually have pneumonia. Furthermore, studies in this era started to expand the diagnostic testing repertoire to include tests of nasopharyngeal secretions and serological assays. The interpretation of these testing methods was often challenging. Regardless, viruses and bacteria were found in a large proportion of cases using these techniques, with most studies focused on a single pathogen of interest [13, 17–20].

### Identification of Multiple Pathogens

In 1983, the Board of Science and Technology for International Development (BOSTID) at the National Academy of Sciences, United States, commissioned a study of acute respiratory infection (ARI) (rather than “pneumonia”) etiology in 10 centers from developing countries [21]. Viruses were cultured from nasopharyngeal aspirates, and bacteria were detected in blood and pleural fluid by culture and in urine by counter-immunoelectrophoresis [22]. Although the BOSTID study had a core protocol, this was adapted at the different study sites, resulting in a variety of definitions of ARI, some of which included cases with upper respiratory infections. Not surprisingly, case-fatality ratios were low due to the inclusion of less severe cases. Lung aspirates were not performed. Viruses were recovered more frequently than bacteria, and detection of multiple potential pathogens in individual patients was common. Respiratory syncytial virus was the most common virus detected, and *S. pneumoniae* and *H. influenzae* were the most commonly detected bacteria. Although the BOSTID studies struggled with how to interpret and present their findings of multiple pathogens, the studies raised the possibility of the synergistic roles of viruses and bacteria in the pathogenesis of ARI.

To capture evidence on a greater number of potential pathogens, pneumonia studies since the BOSTID era have tested a wider range of clinical specimens with an increasing array of methods. For example, a 2002 study of human immunodeficiency virus (HIV)–infected and uninfected children in Durban obtained blood cultures, nasopharyngeal aspirates, induced sputum, gastric washings (for *Mycobacterium tuberculosis*), pleural fluid, and nonbronchoscopic bronchoalveolar lavage fluid [16]. Of 308 children with a complete set of specimens, 141 (46%), 53 (17%), and 4 (1%) had 1, 2, and 4 pathogens identified, respectively [16].

Over the last 15 years there have been tremendous advances in the detection of microorganisms through nucleic acid detection techniques [23]. It is now possible to run high-throughput polymerase chain reaction (PCR) panels that can simultaneously detect very low levels of multiple bacterial and viral targets. Not surprisingly, studies using these multiplex PCR panels to test nasopharyngeal specimens have frequently detected multiple potential pathogens within individual cases [24–27]. Combining many tests, most with imperfect clinical specificity, leads to an accumulation of false-positive results, creating a background noise from which it is difficult to discern the true signals of pneumonia etiology. As is discussed in a companion article [28], it is not until very recently that studies have also sampled nonpneumonia controls contemporaneously with pneumonia cases; case–control studies permit an assessment of the strength of association between a positive test and clinical pneumonia, but the resultant odds ratio is not readily interpretable for causality [24, 25, 27, 29].

### Vaccine Probe Studies

If a pathogen-specific intervention can prevent pneumonia, it provides strong evidence for that pathogen’s role in causing pneumonia, although it may not be an exclusive role. This is the rationale behind vaccine probe studies [30]. For example, in a randomized controlled trial of the conjugate *H. influenzae* type B (Hib) vaccine in The Gambia, the risk of radiologically confirmed pneumonia was 21% lower among vaccine recipients than controls [31]. This provides evidence that at least 21% of cases of pneumonia, whose etiology is unknown, were “caused by” Hib. The true fraction is likely to be even greater because the efficacy of the vaccine against Hib pneumonia is almost certainly <100% [30]. Such studies have confirmed and quantified the dominance of Hib and pneumococcus as causes of radiologically confirmed pneumonia in children from several continents. However, the proportion of cases of clinically defined pneumonia prevented by Hib or pneumococcal vaccines is much smaller because this definition is less specific for true lung infection [30, 32–36]. The vaccine probe technique also has the potential to explore causal pathways and pathogen interactions in pneumonia. For example, it can be used to test whether vaccines against influenza are able to reduce the subsequent incidence of pneumococcal pneumonia. Furthermore, the probe need not be a vaccine, as demonstrated recently by the use of prophylactic monoclonal antibody infusions for the prevention of hospitalizations and outpatient visits caused by respiratory syncytial virus [33].

**Figure 1.**
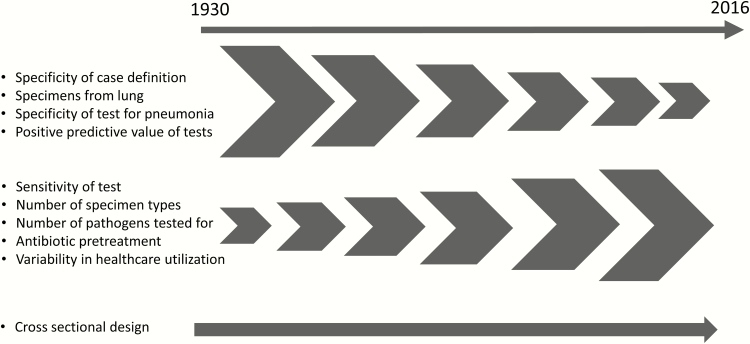
Changes in challenges to pneumonia etiology studies over time, 1930–2016.

The history of pneumonia etiology studies over the last century shows several trends: from the use of highly specific tests on specimens from the lung itself to highly sensitive tests on samples of body fluids distant from the lung; from detection of single pathogens to detection of multiple pathogens; and from an exclusive focus on bacteria to enhanced detection of viruses (Figure 1). Although some challenges facing early investigators (eg, poor viral diagnostics) have improved, others have endured (eg, variable case definitions) or worsened (eg, lack of lung tissue). We now discuss the major challenges hampering pneumonia etiology research today, appreciating the history of these current challenges.

## CHALLENGES IN CURRENT PNEUMONIA ETIOLOGY STUDIES

### Case Definitions Defining Different Syndromes

Unlike case definitions for acute gastroenteritis and febrile illness that are based on the presence of a specific symptom (eg, diarrhea) or sign (eg, fever), case definitions for pneumonia define a syndrome that is irreducible to a single, or even a constellation of, signs and/or symptoms. As already mentioned, WHO created a standardized clinical case management definition for children with suspected pneumonia in the 1980s, placing a priority on sensitivity rather than the specificity achieved in earlier radiologically based definitions [15]. Throughout this article, we refer to sensitivity of a case definition (or test) as the percentage of all children with pneumonia who are identified. On the other hand, specificity refers to the percentage of all children without pneumonia who do not meet a case definition (or did not have a positive test result). High sensitivity usually comes at the expense of misdiagnosing some children without pneumonia as having pneumonia (false positives); high specificity usually comes at the expense of missing some children who have pneumonia (false negatives). Although the WHO’s sensitive case definition was developed to maximize the likelihood that children with pneumonia would be treated with antibiotics in peripheral health facilities that lack radiologic capacity, it has been co-opted for use in many pneumonia etiology and disease burden studies. Using the WHO case management definitions results in misclassification, an acceptable consequence in the clinical setting, but a problematic one in the research setting. Many children with conditions other than pneumonia (eg, sepsis, malaria, upper respiratory tract infections) will be included as pneumonia cases; the pathogens detected in these children will therefore be falsely ascribed as causing pneumonia. Another problem is the variety of pneumonia case definitions used in etiology studies. In a review of pneumonia etiology studies done since the year 2000, 61% of the 153 studies used WHO clinical case definitions. However, even among these studies, not all chose the same definition, with some including definitions for “very severe pneumonia,” some “severe pneumonia,” and some “nonsevere pneumonia” [37]. Half also required the presence of a chest radiographic abnormality, whereas others required evidence of acute infection (eg, fever, leukocytosis) [38]. Just under half of the studies included children with wheezing, which is likely to include more cases with viral infections. Some studies side-stepped all of these rule-based definitions by using a definition of “physician-diagnosed pneumonia” [39]. Such heterogeneity in case definitions leads to incomparability of etiology results.

### Not All Cases Sampled, Especially Fatal Cases

A study may not accurately represent the full distribution of pneumonia etiologies if certain types of patients or certain types of specimens are investigated less frequently. For example, if there is a likelihood of collecting body fluid samples more commonly from more severe cases, this biases etiologic distributions toward those more likely to cause severe pneumonia. A more entrenched limitation, however, is that specimens from fatal pneumonia cases are underrepresented in etiology studies. Fatal cases likely have a different etiologic pattern than nonfatal cases, and studies solely focused on the latter will not accurately represent the causes of fatal pneumonia.

There are several reasons for the underrepresentation of fatal cases in pneumonia etiology studies. First, in many settings where healthcare utilization is poor, the sickest children die before presentation to the hospital, and few studies have investigated etiology among cases identified outside of health facilities. At the time of presentation to the hospital, the most critically ill cases are often not enrolled in etiology studies due to the urgent need for resuscitation and the reluctance to perform research procedures perceived as potentially adversely affecting the child’s precarious clinical condition. Moreover, the sickest children often die soon after presentation before they can be enrolled or before specimens can be collected. Postmortem specimens are rarely collected. In a pneumonia etiology study in Kilifi, Kenya, children who met eligibility criteria but who were not enrolled had a case-fatality ratio of 18% compared with 4% among those enrolled, illustrating the survivorship bias [26]. Researchers are left to extrapolate the causes of fatal pneumonia from the most severe cases enrolled, which leads to uncertainty about the true causes of pneumonia mortality.

### Not All Specimen Types Collected

The reported etiologies of pneumonia are strongly influenced by the types of clinical specimens collected. Sterile-site specimens have been the gold standard for detection of bacterial pneumonia, although their poor sensitivity is well established. Upper respiratory tract samples will detect both viruses and bacteria, although their etiologic significance is questionable. Some pathogens are preferentially identified in oropharyngeal swabs, compared with nasopharyngeal swabs, such as *Mycoplasma pneumoniae* [40]. Tuberculosis is most often diagnosed in children by testing induced sputum or gastric aspirates. Pneumocystis pneumonia is most definitively identified by bronchoalveolar lavage or induced sputum. A review of published pneumonia etiology studies from 2000–2010 revealed that 77% collected blood and 15% collected pleural fluid [37]. Upper respiratory tract specimens were collected in approximately half of studies; only 10% collected induced sputum.

### Likelihood of Seeking Care for Pneumonia Differs by Site

Healthcare utilization practices vary widely around the world [41]. In some cultures, parents seek care early, particularly for young children, whereas in other settings, parents seek remedies for their child’s mild illness at a traditional healer and only present to hospital if this approach fails. In some low-income countries, access is also limited by distance, cost, or time considerations [42]. In such settings, children often present late in the course of illness, when their clinical status has become severe or even moribund [43]. In studies of children who present early in the course of illness, the contribution of pathogens that cause mild or moderate pneumonia, such as some viruses, will dominate. In contrast, in studies of children who arrive at hospital late in the course of illness, the etiologic spectrum will reflect pathogens causing severe pneumonia, particularly bacteria. Although pneumonia etiology studies at both extremes might accurately represent the causes of hospitalized pneumonia in each setting, they are not necessarily describing the same clinical syndrome or the full etiologic spectrum of pneumonia in the community.

### Cross-Sectional Designs Cannot Describe the Causal Chain of Pneumonia

The majority of pneumonia etiology studies are cross-sectional in design, whereby specimens are collected at the time of admission or presentation to a health facility. Sampling at 1 point in time will fail to detect the causative pathogen if that pathogen has already been cleared from the sample (eg, bacteria in the blood). Moreover, cross-sectional designs provide little information on the causal chain of pneumonia or the synergistic role of multiple pathogens in causation. There is considerable evidence that influenza virus can damage the respiratory epithelial cells, making a person susceptible to a subsequent bacterial pneumonia [44–47]. Other viruses, such as parainfluenza virus and adenovirus, have also been implicated as playing a causal role in subsequent bacterial pneumonia [48, 49].

### Specimens Distant From the Site of Infection

Lung aspirates are now rarely performed in either clinical practice or pneumonia etiology studies. Pneumonia diagnosis now relies on findings from specimens indirectly from or peripheral to the site of infection, such as blood, induced sputum, nasopharyngeal and oropharyngeal secretions, gastric aspirates, or urine [50–52].

Samples not obtained directly from the lung pose problems of both sensitivity and specificity in assigning pneumonia etiology. A positive bacterial culture in the blood of a patient with clinical pneumonia is widely accepted to indicate pneumonia etiology. However, the blood culture is only positive in a small fraction (approximately 10%) of true bacterial pneumonia cases, making blood culture an insensitive diagnostic test [32]. Secretions from the lower respiratory tract of children with pneumonia offer diagnostic promise because of their origin in the lung and their ability to be collected in a noninvasive manner (ie, induced sputum). However, the inferential value of this specimen is critically dependent on the collection of a true lower respiratory tract specimen free of contamination by upper respiratory tract secretions, an outcome difficult to achieve [53].

Upper respiratory tract specimens pose a particular problem in pneumonia diagnostics. Because these specimens are easy to obtain, they are now commonly used to assess and infer the cause of pneumonia [37]. Polymerase chain reaction of upper respiratory tract specimens has high sensitivity but low specificity for establishing pneumonia etiology for most pathogens [23, 29]. Because most viruses that can cause pneumonia more often cause upper respiratory tract infections, detection of a virus in the upper respiratory tract of a pneumonia patient might only represent infection of the upper respiratory tract. Moreover, detection of viral nucleic acid might indicate asymptomatic infection or prolonged shedding from a resolved illness episode rather than current symptomatic infection. Detecting some common bacteria (eg, pneumococcus, *Moraxella catarrhalis*) in the upper respiratory tract often depicts a state of commensal colonization rather than illness. In many developing country settings, pneumococci can be found in the nasopharynx of almost all children, regardless of the presence of symptoms [54]. Strategies such as quantification of pathogen load, strain identification, and assessment of attributable fraction have been used to overcome the specificity problem of upper respiratory specimens and are described elsewhere [28, 29, 55, 56].

### Antibiotic Pretreatment

Microbiologic diagnosis of the cause of pneumonia is also hampered by the frequent use of outpatient antibiotics, which are available without a prescription in some locations. Antibiotic pretreatment further decreases the sensitivity of bacterial cultures [16]. The magnitude of this effect is not well quantified and likely varies depending on the type of antibiotic, the duration of antibiotic use prior to specimen collection, and the susceptibility of the pathogen. Regardless, antibiotic use prior to specimen collection leads to an underestimation of the proportion of pneumonia cases attributed to bacterial causes. Designing etiology studies in a way that accounts for or adjusts for this effect is challenging because accurate information on antibiotic pretreatment is difficult to obtain. Parental history is unreliable, bioassays of serum are insensitive because of the high rate of clearance from the serum, and timely urine specimens are difficult to obtain from ill, often dehydrated, children.

### Variability in Method and Number of Pathogens Tested

The findings on etiologic distribution of pneumonia are dependent on which pathogens are included in the testing panel. Obviously, etiology cannot be attributed to a pathogen not tested for. Testing for only 1 or 2 pathogens will overestimate the causal role of these pathogens because studies usually assign complete causal attribution when detected. On the other hand, addition of multiple tests with less than perfect specificity for many pathogens, particularly of low prevalence as true causes of pneumonia, will lead to a greater likelihood of false-positive results and the sharing of causal attribution between true pneumonia-causing pathogens and those of less clear etiologic significance. Although bacterial culture does not require investigators to limit the number of bacteria tested for, using PCR in etiology studies dictates an a priori list of putative pathogens, which might include some pathogens that actually do not cause pneumonia while excluding others that do. Nontargeted detection methods (eg, metagenomics) avoid the latter problem but raise further dilemmas of interpretability of multiple pathogen detection in nonsterile sites [57, 58].

The picture of etiology is also determined by the performance characteristics of the assays used. For bacterial culture, the choice of and quality of media substantially influences the pathogens that are detectable and can lead to incorrect conclusions on pathogen prevalence (eg, appropriate blood agar for pneumococcus). Polymerase chain reaction assay performance can vary for the same pathogen [59]. Measurement error is difficult to estimate and is therefore rarely incorporated into the analysis of etiology.

### Multiple Pathogens

Although Occam’s razor favors the hypothesis with the fewest assumptions (eg, that a pneumonia episode is caused by a single pathogen), in the case of pneumonia, biology seems reluctant to comply with this premise. There is abundant evidence that viral infections can predispose an individual to bacterial pneumonia [47, 48]. Yet, assigning multiple pathogens as the cause of pneumonia remains a methodologic and analytic challenge. Some pathogens might play a role early in the course of pneumonia and be gone from the sampled body site by the time the pneumonic process manifests. On the other hand, highly sensitive assays of the upper respiratory tract can identify multiple pathogens of unclear etiologic significance in the same individual. Even finding >1 pathogen in what is usually considered a sterile site, like blood, does not assure that each is playing a causal role in the lung infection. Moreover, recent evidence from non-culture-based detection methods has challenged the long-standing notion that the lung itself is a sterile site; the healthy lung likely has its own microbiome and might also experience the transient presence of putative pneumonia pathogens, perhaps through microaspiration of upper respiratory tract flora, of unclear pathophysiologic significance [57, 58, 60, 61].

Faced with the challenge of attributing etiology, some researchers report all combinations of pathogens detected in pneumonia cases [21, 38]. Although true to the data, this option results in a long list of pathogen combinations that does not lend itself to a clear understanding of actual etiology or to optimal treatment and prevention strategies [38, 62]. Analytic approaches that attempt, in part, to assign population-level and individual-level causality to each pathogen have been developed and are described in more detail in another article in this supplement [28].

## AVERTING THE CHALLENGES IN FUTURE ETIOLOGY STUDIES

There have been many technological and methodological advances since the days of culturing pneumococcus for the purpose of horse serum therapy. Although many more pathogens can now be detected and modern assays have substantially higher sensitivity than older tests, the specimens most commonly sampled now are less directly related to the site of infection than the lung aspirates used in earlier decades. This evolution in specimens collected, methods for testing, and range of pathogens tested for poses a challenge of integrating a plethora of data of variable accuracy into an analysis from which meaningful biologic inferences can be drawn about etiology.

We suggest several approaches and needs for the future of pneumonia etiology studies.

1. Lung aspirates should be formally evaluated, and if found to be safe and beneficial, considered for wider use. Lung material remains the most useful specimen because it is a sample from the site of infection. Lung aspirate procedures have been shown to have a good safety profile in well-trained hands, yield results that can improve acute care for the individual patient, and have high value for pneumonia etiology studies. Several research groups have recently returned to this gold-standard diagnostic and applied new molecular diagnostic assays to sampled lung tissue. These studies show the presence of multiple pathogens in the lungs of children with pneumonia [63, 64]. As mentioned, current thinking no longer holds the lung to be a sterile site, complicating interpretation of finding pathogens even in the lung. Clinical outcomes of those who have undergone lung aspirate should be documented and, if possible, compared with outcomes of similar patients who did not undergo the procedure to evaluate whether lung aspirates either harmed (eg, excess pneumothoraces) or benefited (eg, more targeted antibiotic therapy) a population of pneumonia patients. If found to be safe and beneficial, consideration should be given to expand the collection of lung aspirates and to possibly extend their collection to a broader distribution of pneumonia cases, beyond those with a large, peripheral, consolidated infiltrate on chest radiograph.
2. Pneumonia etiology studies should prioritize the examination of fatal cases. Postmortem evaluations, especially limited to examination of the chest, and collection of nasopharyngeal and blood specimens in the immediate postmortem period are likely to be highly informative [65]. An autopsy study of 290 Zambian children with a clinical diagnosis of pneumonia in 2002 diagnosed pyogenic pneumonia in approximately half of all cases but also found a sizeable number of cases with other pathology, such as pulmonary edema and shock lung, indicative of clinical misclassification during life [66]. Surprisingly, a quarter of all HIV-uninfected children had tuberculosis. Although postmortem samples can add to our knowledge, they have their own set of limitations, such as high rates of refusal, lack of clarity regarding initial etiology among cases with prolonged hospital courses, and contamination by postmortem bacterial overgrowth. The use of autopsies, including minimally invasive studies, are now the centerpiece of a new initiative to determine the cause of death in a network of surveillance sites in Africa and Asia [67, 68]. Because postmortem pneumonia studies by definition exclude surviving severe pneumonia cases, their contribution to describing severe pneumonia etiology should be complemented with data from the majority of studies that describe predominantly nonfatal pneumonia cases.
3. Pneumonia etiology studies should use case definitions based on radiologic evidence. In studies of pneumonia etiology, the shift toward application of the WHO clinical case management definitions has led to misclassification of other respiratory and nonrespiratory illnesses as pneumonia. The proportion of misclassified cases can be substantial, and assignment of causality to these cases can result in inaccurate etiologic determinations, leading to misguided clinical or public health interventions. Evidence of lung parenchymal involvement on chest radiograph, although imperfect, is the most accurate and accessible indicator of pneumonia. Although clinical case definitions still have a role in clinical management, efforts should be made to characterize the nature and etiology of the nonpneumonia illnesses captured by those definitions.
4. We need to develop a better understanding of the pathogenesis of pneumonia. The causal chain of pneumonia and the role of multiple pathogens in that chain remain a refractory enigma. Basic questions remain unresolved. Can viruses cause severe pneumonia on their own? Can bacteria cause pneumonia without a preceding viral infection? What host-related factors enable a pathogen or multiple pathogens to cause pneumonia and in what sequence? Why does a dominant species emerge from the lung ecosystem in pneumonia? Is there a set of immunologic responses to microbiota that distinguishes asymptomatic infection from disease, and if so, are these responses specific enough for certain pathogens to be used diagnostically [69]? The vast majority of past pneumonia etiology studies used a cross-sectional design that is unable to answer these questions. Prospective studies with recurrent longitudinal sampling are resource-intensive, underpowered to detect a rare outcome like pneumonia, and still susceptible to unclear interpretation of pathogen detection. Therefore insights into pathogenesis might be most likely found in the controlled experimental conditions of animal studies. The vaccine probe approach, which yielded insights into the etiologic fraction of pneumonia caused by Hib and pneumococcus [30], can potentially be extended further in clarifying the causal direction of relationships between pathogens causing disease. Although probe studies provide strong evidence of causality, they are limited by the small number of highly effective pathogen-specific interventions available [30]. Ultimately, a better understanding of how pneumonia occurs can direct the types of tests we do and how they are interpreted with regard to pneumonia etiology.

Over the past century of pneumonia etiology studies, we have seen that as some challenges are resolved, others arise. For example, as the focus moved from pneumococcus only to multiple pathogens detected in sites distant from the lung, the attribution of etiology became more complex. Alternatively, some challenges exacerbate others. The use of a nonspecific clinical case definition in pneumonia etiology studies made the interpretation of nonspecific tests (eg, PCR of nasopharyngeal specimens) more troublesome. History also demonstrates that no single study is able to solve all of the challenges of pneumonia etiology. More likely, the most comprehensive picture of pneumonia etiology will need to come from piecing together different, complementary studies, such as cross-sectional studies, postmortem studies, probe studies, lung aspirate studies, and animal models. Ultimately, efforts to overcome the challenges of pneumonia etiology studies of the past could have meaningful impact in pneumonia treatment and prevention in the future.

## Supplementary Data

Supplementary materials are available at *Clinical Infectious Diseases* online. Consisting of data provided by the authors to benefit the reader, the posted materials are not copyedited and are the sole responsibility of the authors, so questions or comments should be addressed to the corresponding author.

## Supplementary Material

OTH1_SupplementalMaterial

## Notes

***Author contributions.*** D. R. F. and J. A. G. S. drafted the initial manuscript. L. L. H., D. R. M., and K. L. O. provided significant contributions to the development of the manuscript. All authors reviewed and approved the manuscript.

***Acknowledgments.*** The authors acknowledge the significant contributions of the PERCH Study Group and all PERCH investigators. The authors offer their gratitude to the members of the Pneumonia Methods Working Group and PERCH Expert Group for their time and lending expertise to assist the PERCH Study Group. See the Supplementary Materials for a full list of names. This paper is published with the permission of the director of the Kenya Medical Research Institute.

***Financial support.*** PERCH was supported by grant 48968 from the Bill & Melinda Gates Foundation to the International Vaccine Access Center, Department of International Health, Johns Hopkins Bloomberg School of Public Health. J. A. G. S. was supported by a clinical fellowship from The Wellcome Trust of Great Britain (098532).

***Supplement sponsorship.*** This article appears as part of the supplement “Pneumonia Etiology Research for Child Health (PERCH): Foundational Basis for the Primary Etiology Results,” sponsored by a grant from the Bill & Melinda Gates Foundation to the PERCH study of Johns Hopkins Bloomberg School of Public Health, Baltimore, Maryland.

***Potential conflicts of interest.*** L. L. H. has received grant funding from Pfizer and GlaxoSmithKline. K. L. O. has received grant funding from GSK and Pfizer and participates on technical advisory boards for Merck, Sanofi Pasteur, PATH, Affinivax, and ClearPath. All other authors: No reported conflicts. All authors have submitted the ICMJE Form for Disclosure of Potential Conflicts of Interest. Conflicts that the editors consider relevant to the content of the manuscript have been disclosed.

## References

[1] Pasteur L Sur une maladie nouvelle, provoquée par la salive d’un enfant mort de la rage. Bull L’Académie médecine. 1881; 92:159.

[2] Sternberg G. A fatal form of septicaemia in the rabbit, produced by subcutaneous injection of human saliva: an experimental research. National Board of Health Annual Report Washington, DC: John Murphy & Co., 1881.

[3] Friedländer C Ueber die Schizomyceten bei der akuten fibrösen Pneumonie. Virchows Arch f Path Anat 1882; 8:319.

[4] Friedländer C Die mikrokokken der pneumonie. Fortchr d Med 1883; 1:715.

[5] Leyden. Verhandlungen des vereins für innere medicin. Ueber infectiöse pneumonie. Deutsch Med Wchnschr 1883; 9:52–4.

[6] Talamon M Coccus de la pneumoniae. Bull la Société Anat Paris 1883; 63:475–81.

[7] Foster W. A history of medical bacteriology and immunology. London: Butterworth-Heinemann, 1970.

[8] Cecil R Baldwin H Larsen N Clinical and bacteriologic study of two thousand typed cases of lobar pneumonia. Trans Assoc Am Physicians 1926; 41:208–23.

[9] Sutliff W Finland M The significance of the newly classified types of pneumococci in disease: Types IV to XX inclusive. JAMA 1933; 101:1289–94.

[10] Bullowa J. The management of the pneumonias: for physicians and medical students. New York: Oxford University Press, 1937.

[11] Hansman D Bullen M A resistant pneumococcus. Lancet 1967; 290:264–265. 10.1016/s0140-6736(75)91547-0 48665

[12] Wall RA Corrah PT Mabey DC Greenwood BM The etiology of lobar pneumonia in The Gambia. Bull World Health Organ 1986; 64:553–8. 3490924 PMC2490896

[13] Shann F Walters S Pifer L Pneumonia associated with infection with pneumocystis, respiratory syncytial virus, chlamydia, mycoplasma, and cytomegalovirus in children in Papua New Guinea. Br Med J (Clin Res Ed) 1986; 292:314–7. 10.1136/bmj.292.6516.314 PMC1339284 3002538

[14] Bang AT Bang RA Tale O Reduction in pneumonia mortality and total childhood mortality by means of community-based intervention trial in Gadchiroli, India. Lancet 1990; 336:201–6. 1973770 10.1016/0140-6736(90)91733-q

[15] World Health Organization. Technical bases for the WHO recommendations on the management of pneumonia in children at first-level health facilities: Programme for the Control of Acute Respiratory Infections. Geneva: World Health Organization, 1991.

[16] McNally LM Jeena PM Gajee K Effect of age, polymicrobial disease, and maternal HIV status on treatment response and cause of severe pneumonia in South African children: a prospective descriptive study. Lancet 2007; 369:1440–51. 17467514 10.1016/S0140-6736(07)60670-9

[17] Avendaño LF Larrañaga C Palomino MA Community- and hospital-acquired respiratory syncytial virus infections in Chile. Pediatr Infect Dis J 1991; 10:564–8. 1891287 10.1097/00006454-199108000-00003

[18] Heiskanen-Kosma T Korppi M Laurila A Jokinen C Kleemola M Saikku P *Chlamydia pneumoniae* is an important cause of community-acquired pneumonia in school-aged children: serological results of a prospective, population-based study. Scand J Infect Dis 1999; 31:255–9. 10482053 10.1080/00365549950163536

[19] Lehmann D Sanders RC Marjen B High rates of *Chlamydia trachomatis* infections in young Papua New Guinean infants. Pediatr Infect Dis J 1999; 18(suppl 10):S62–9. 10530576 10.1097/00006454-199910001-00011

[20] Madhi SA Ludewick H Abed Y Klugman KP Boivin G Human metapneumovirus-associated lower respiratory tract infections among hospitalized human immunodeficiency virus type 1 (HIV-1)-infected and HIV-1-uninfected African infants. Clin Infect Dis 2003; 37:1705–10. 14689355 10.1086/379771 PMC7109767

[21] Selwyn BJ The epidemiology of acute respiratory tract infection in young children: comparison of findings from several developing countries. Coordinated Data Group of BOSTID Researchers. Rev Infect Dis 1990; 12(suppl 8:S870–88. 2270410 10.1093/clinids/12.supplement_s870

[22] Bale JR Creation of a research program to determine the etiology and epidemiology of acute respiratory tract infection among children in developing countries. Rev Infect Dis 1990; 12(suppl 8):S861–6. 10.1093/clinids/12.supplement_8.s861 2270408

[23] Murdoch DR O’Brien KL Scott JA Breathing new life into pneumonia diagnostics. J Clin Microbiol 2009; 47:3405–8. 19741073 10.1128/JCM.01685-09 PMC2772647

[24] Berkley JA Munywoki P Ngama M Viral etiology of severe pneumonia among Kenyan infants and children. JAMA 2010; 303:2051–7. 20501927 10.1001/jama.2010.675 PMC2968755

[25] Feikin D Njenga M Bigogo G Etiology and incidence of viral and bacterial acute respiratory illness among older children and adults in rural western Kenya, 2007–2010. PLoS One 2012; 7:e43656. 22937071 10.1371/journal.pone.0043656 PMC3427162

[26] Hammitt LL Kazungu S Morpeth SC A preliminary study of pneumonia etiology among hospitalized children in Kenya. Clin Infect Dis 2012; 54 suppl 2:S190–9. 22403235 10.1093/cid/cir1071 PMC3297554

[27] Singleton RJ Bulkow LR Miernyk K Viral respiratory infections in hospitalized and community control children in Alaska. J Med Virol 2010; 82:1282–90. 20513097 10.1002/jmv.21790 PMC7167028

[28] Hammitt LL, Feikin DR, Scott JAG Addressing the analytic challenges of cross-sectional pediatric pneumonia etiology data. Clin Infect Dis 2017; 64(suppl 3): S197–204. 10.1093/cid/cix147 PMC5447845 28575372

[29] Self WH Williams DJ Zhu Y Respiratory viral detection in children and adults: comparing asymptomatic controls and patients with community-acquired pneumonia. J Infect Dis 2016; 213:584–91. 26180044 10.1093/infdis/jiv323 PMC4721902

[30] Feikin DR Scott JA Gessner BD Use of vaccines as probes to define disease burden. Lancet 2014; 383:1762–70. 24553294 10.1016/S0140-6736(13)61682-7 PMC4682543

[31] Mulholland K Hilton S Adegbola R Randomised trial of *Haemophilus influenzae* type-b tetanus protein conjugate vaccine for prevention of pneumonia and meningitis in Gambian infants. Lancet 1997; 349:1191–7. 9130939 10.1016/s0140-6736(96)09267-7

[32] Cutts FT Zaman SM Enwere G Efficacy of nine-valent pneumococcal conjugate vaccine against pneumonia and invasive pneumococcal disease in The Gambia: randomised, double-blind, placebo-controlled trial. Lancet 2005; 365:1139–46. 15794968 10.1016/S0140-6736(05)71876-6

[33] O’Brien KL Chandran A Weatherholtz R Efficacy of motavizumab for the prevention of respiratory syncytial virus disease in healthy Native American infants: a phase 3 randomised double-blind placebo-controlled trial. Lancet Infect Dis 2015; 15:1398–408. 26511956 10.1016/S1473-3099(15)00247-9

[34] Gessner BD Sutanto A Linehan M Incidences of vaccine-preventable *Haemophilus influenzae* type B pneumonia and meningitis in Indonesian children: hamlet-randomised vaccine-probe trial. Lancet 2005; 365:43–52. 15643700 10.1016/s0140-6736(04)17664-2

[35] Levine OS Lagos R Muñoz A Defining the burden of pneumonia in children preventable by vaccination against *Haemophilus influenzae* type B. Pediatr Infect Dis J 1999; 18:1060–4. 10608624 10.1097/00006454-199912000-00006

[36] Klugman KP Madhi SA Huebner RE Kohberger R Mbelle N Pierce N; Vaccine Trialists Group A trial of a 9-valent pneumococcal conjugate vaccine in children with and those without HIV infection. N Engl J Med 2003; 349:1341–8. 14523142 10.1056/NEJMoa035060

[37] Gilani Z Kwong YD Levine OS A literature review and survey of childhood pneumonia etiology studies: 2000–2010. Clin Infect Dis 2012; 54 suppl 2:S102–8. 22403223 10.1093/cid/cir1053 PMC3693495

[38] Olsen SJ Thamthitiwat S Chantra S Incidence of respiratory pathogens in persons hospitalized with pneumonia in two provinces in Thailand. Epidemiol Infect 2010; 138:1811–22. 20353622 10.1017/S0950268810000646

[39] Moyes J Cohen C Pretorius M Epidemiology of respiratory syncytial virus-associated acute lower respiratory tract infection hospitalizations among HIV-infected and HIV-uninfected South African children, 2010–2011. J Infect Dis 2013; 208(suppl 3):S217–26. 24265481 10.1093/infdis/jit479

[40] Loens K Van Heirstraeten L Malhotra-Kumar S Goossens H Ieven M Optimal sampling sites and methods for detection of pathogens possibly causing community-acquired lower respiratory tract infections. J Clin Microbiol 2009; 47:21–31. 19020070 10.1128/JCM.02037-08 PMC2620840

[41] Deutscher M Beneden CV Burton D Putting surveillance data into context: the role of health care utilization surveys in understanding population burden of pneumonia in developing countries. J Epidemiol Glob Health 2012; 2:73–81. 23856423 10.1016/j.jegh.2012.03.001 PMC7103994

[42] Noor AM Zurovac D Hay SI Ochola SA Snow RW Defining equity in physical access to clinical services using geographical information systems as part of malaria planning and monitoring in Kenya. Trop Med Int Health 2003; 8:917–26. 14516303 10.1046/j.1365-3156.2003.01112.x PMC2912492

[43] Moïsi JC Gatakaa H Noor AM Geographic access to care is not a determinant of child mortality in a rural Kenyan setting with high health facility density. BMC Public Health 2010; 10. 10.1186/1471-2458-10-142 PMC2848200 20236537

[44] Hament JM Kimpen JL Fleer A Wolfs TF Respiratory viral infection predisposing for bacterial disease: a concise review. FEMS Immunol Med Microbiol 1999; 26(3-4):189–95. 10575129 10.1111/j.1574-695X.1999.tb01389.x

[45] Shann F Gratten M Germer S Linnemann V Hazlett D Payne R Aetiology of pneumonia in children in Goroka Hospital, Papua New Guinea. Lancet 1984; 2:537–41. 6147602 10.1016/s0140-6736(84)90764-5

[46] Klugman KP Madhi SA Pneumococcal vaccines and flu preparedness. Science 2007; 316:49–50. 10.1126/science.316.5821.49c 17412937

[47] McCullers JA Insights into the interaction between influenza virus and pneumococcus. Clin Microbiol Rev 2006; 19:571–82. 16847087 10.1128/CMR.00058-05 PMC1539103

[48] Fiore AE Iverson C Messmer T Outbreak of pneumonia in a long-term care facility: antecedent human parainfluenza virus 1 infection may predispose to bacterial pneumonia. J Am Geriatr Soc 1998; 46:1112–7. 9736104 10.1111/j.1532-5415.1998.tb06649.x

[49] Feikin DR Moroney JF Talkington DF An outbreak of acute respiratory disease caused by *Mycoplasma pneumoniae* and adenovirus at a federal service training academy: new implications from an old scenario. Clin Infect Dis 1999; 29:1545–50. 10585810 10.1086/313500

[50] Murdoch DR O’Brien KL Driscoll AJ Karron RA Bhat N; Pneumonia Methods Working Group; PERCH Core Team Laboratory methods for determining pneumonia etiology in children. Clin Infect Dis 2012; 54(suppl 2):S146–52. 22403229 10.1093/cid/cir1073

[51] Hammitt LL Murdoch DR Scott JA; Pneumonia Methods Working Group. Specimen collection for the diagnosis of pediatric pneumonia. Clin Infect Dis 2012; 54(suppl 2):S132–9. 22403227 10.1093/cid/cir1068 PMC3693496

[52] Clark JE Determining the microbiological cause of a chest infection. Arch Dis Child 2015; 100:193–7. 25246089 10.1136/archdischild-2013-305742

[53] Thea DM Hammitt LL Seidenberg P Limited utility of PCR on induced sputum for diagnosing the etiology of childhood pneumonia in resource poor settings: findings from the Pneumonia Etiology Research for Child Health (PERCH) Study. Clin Infect Dis 2017; 64(suppl 3):S289–300. 10.1093/cid/cix098 PMC5447848 28575363

[54] Hill PC Townend J Antonio M Transmission of *Streptococcus pneumoniae* in rural Gambian villages: a longitudinal study. Clin Infect Dis 2010; 50:1468–76. 20420503 10.1086/652443

[55] Feikin DR, Fu W Park DE Is higher viral load in the upper respiratory tract associated with severe pneumonia? Findings from the PERCH study. Clin Infect Dis 2017; 64(suppl 3):S337–46. 10.1093/cid/cix148 PMC5447843 28575373

[56] Park DE, Baggett HC Howie SRC Colonization density of the upper respiratory tract as a predictor of pneumonia—*Haemophilus influenzae, Moraxella catarrhalis, Staphylococcus aureus*, and *Pneumocystis jirovecii*. Clin Infect Dis 2017; 64(suppl 3):S328–36. 10.1093/cid/cix104 PMC5612712 28575367

[57] Beck JM ABCs of the lung microbiome. Ann Am Thorac Soc 2014; 11(suppl 1):S3–6. 24437402 10.1513/AnnalsATS.201306-188MG PMC3972977

[58] Segal LN Rom WN Weiden MD Lung microbiome for clinicians. New discoveries about bugs in healthy and diseased lungs. Ann Am Thorac Soc 2014; 11:108–16. 24460444 10.1513/AnnalsATS.201310-339FR PMC3972985

[59] Driscoll AJ Karron RA Bhat N Evaluation of fast-track diagnostics and TaqMan array card real-time PCR assays for the detection of respiratory pathogens. J Microbiol Methods 2014; 107:222–6. 25448378 10.1016/j.mimet.2014.10.009 PMC7114243

[60] Dickson RP Erb-Downward JR Martinez FJ Huffnagle GB The microbiome and the respiratory tract. Annu Rev Physiol 2016; 78:481–504. 26527186 10.1146/annurev-physiol-021115-105238 PMC4751994

[61] Dickson RP Erb-Downward JR Huffnagle GB Towards an ecology of the lung: new conceptual models of pulmonary microbiology and pneumonia pathogenesis. Lancet Respir Med 2014; 2:238–46. 24621685 10.1016/S2213-2600(14)70028-1 PMC4004084

[62] Tupasi TE Lucero MG Magdangal DM Etiology of acute lower respiratory tract infection in children from Alabang, Metro Manila. Rev Infect Dis 1990; 12 suppl 8:S929–39. 2270415 10.1093/clinids/12.supplement_8.s929

[63] Carrol ED Mankhambo LA Guiver M PCR improves diagnostic yield from lung aspiration in Malawian children with radiologically confirmed pneumonia. PLoS One 2011; 6:e21042. 21695128 10.1371/journal.pone.0021042 PMC3114850

[64] Howie SR Morris GA Tokarz R Etiology of severe childhood pneumonia in the Gambia, West Africa, determined by conventional and molecular microbiological analyses of lung and pleural aspirate samples. Clin Infect Dis 2014; 59:682–5. 24867789 10.1093/cid/ciu384 PMC4130311

[65] Turner GD Bunthi C Wonodi CB The role of postmortem studies in pneumonia etiology research. Clin Infect Dis 2012; 54 suppl 2:S165–71. 22403232 10.1093/cid/cir1062 PMC3297548

[66] Chintu C Mudenda V Lucas S; UNZA-UCLMS Project Paediatric Post-mortem Study Group. Lung diseases at necropsy in African children dying from respiratory illnesses: a descriptive necropsy study. Lancet 2002; 360:985–90. 12383668 10.1016/S0140-6736(02)11082-8

[67] Martínez MJ Massora S Mandomando I Infectious cause of death determination using minimally invasive autopsies in developing countries. Diagn Microbiol Infect Dis 2016; 84:80–6. 26508103 10.1016/j.diagmicrobio.2015.10.002

[68] The Bill & Melinda Gates Foundation. The Bill & Melinda Gates Foundation to fund disease surveillance network in africa and asia to prevent childhood mortality and help prepare for the next epidemic http://www.gatesfoundation.org/Media-Center/Press-Releases/2015/05/Child-Health-and-Mortality-Prevention-Surveillance-Network Accessed 28 December 2015.

[69] Huang H Ideh RC Gitau E Discovery and validation of biomarkers to guide clinical management of pneumonia in African children. Clin Infect Dis 2014; 58:1707–15. 24696240 10.1093/cid/ciu202 PMC4036688

