# Supplementary material for: The Enduring Challenge of Determining Pneumonia Etiology in Children: Considerations for Future Research Priorities
Source: Clin Infect Dis. 2017 May 27;64(Suppl 3):S188–96. doi: 10.1093/cid/cix143 (PMC5447852; doi:10.1093/cid/cix143)
Supplement: OTH1_SupplementalMaterial [file cix143_suppl_OTH1_SupplementalMaterial.pdf]

Addressing the analytic challenges of cross-sectional pediatric pneumonia etiology data
Supplemental Materials

Acknowledgements:

PERCH Study Group. **Johns Hopkins Bloomberg School of Public Health, Baltimore, Maryland**: Katherine L. O'Brien (PI), Orin S. Levine (Former PI, current affiliation Bill & Melinda Gates Foundation, Seattle, Washington**),** Maria Deloria Knoll (co-PI), Daniel R. Feikin (joint affiliation with Centers for Disease Control and Prevention, Atlanta, Georgia), Andrea N. DeLuca, Amanda J. Driscoll, Nicholas Fancourt, Wei Fu, Laura L. Hammitt, Melissa M. Higdon, E. Wangeci Kagucia, Ruth A. Karron, Mengying Li, Daniel E. Park, Christine Prosperi, Zhenke Wu, Scott L. Zeger; **The Emmes Corporation, Rockville, Maryland:** Nora L. Watson, **Nuffield Department of Clinical Medicine, University of Oxford, United Kingdom**: Jane Crawley; **University of Otago, Christchurch, New Zealand**: David R. Murdoch; **ICDDR, b, Dhaka and Matlab, Bangladesh**: W. Abdullah Brooks (site PI), Hubert P. Endtz, Khalequ Zaman, Doli Goswami, Lokman Hossain, Yasmin Jahan, Hasan Ashraf; **Medical Research Council, Basse, The Gambia**: Stephen R. C. Howie (site PI), Bernard E. Ebruke, Martin Antonio, Jessica McLellan, Eunice Machuka, Arifin Shamsul, Syed M.A. Zaman, Grant Mackenzie; **KEMRI-Wellcome Trust Research Programme, Kilifi, Kenya**: J. Anthony G. Scott (site PI and PERCH co-PI), Juliet O. Awori, Susan C. Morpeth, Alice Kamau, Sidi Kazungu, Micah Silaba Ominde; **Division of Infectious Disease and Tropical Pediatrics, Department of Pediatrics, Center for Vaccine Development, Institute of Global Health, University of Maryland School of Medicine, Baltimore, Maryland and Centre pour le Développement des Vaccins (CVD-Mali), Bamako, Mali**: Karen L. Kotloff (site PI), Milagritos D. Tapia, Samba O. Sow, Mamadou Sylla, Boubou Tamboura, Uma Onwuchekwa, Nana Kourouma, Aliou Toure; **Respiratory and Meningeal Pathogens Research Unit, University of the Witwatersrand, Johannesburg, South Africa**: Shabir A. Madhi (site PI), David P. Moore, Peter V. Adrian, Vicky L. Baillie, Locadiah Kuwanda, Azwifarwi Mudau, Michelle J. Groome, Nasreen Mahomed, **Thailand Ministry of Public Health – U.S. CDC Collaboration, Nonthaburi, Thailand**: Henry C. Baggett (site PI), Somsak Thamthitiwat, Susan A. Maloney (former site PI), Charatdao Bunthi, Julia Rhodes, Pongpun Sawatwong, Pasakorn Akarasewi (site co-PI, Ministry of Public Health); **Boston University School of Public Health, Boston, Massachusetts and University Teaching Hospital, Lusaka, Zambia**: Donald M. Thea (site PI), Lawrence Mwananyanda, James Chipeta, Phil Seidenberg, James Mwansa, Somwe wa Somwe, Geoffrey Kwenda.
